# Impact of point‐of‐care maternal viral load testing at delivery on vertical HIV transmission risk assessment and neonatal prophylaxis: a cluster randomized trial

**DOI:** 10.1002/jia2.70021

**Published:** 2025-07-28

**Authors:** Anange Fred Lwilla, Kira Elsbernd, Siriel Boniface, Raphael Edom, Arlete Mahumane, Bindiya Meggi, W. Chris Buck, Joaquim Lequechane, Kassia Pereira, Nhamo Chiwerengo, Falume Chale, Chishamiso Mudenyanga, Dadirayi Mutsaka, Marianna Mueller, Nyanda E. Ntinginya, Nuno Taveira, Michael Hoelscher, Ilesh Jani, Arne Kroidl, Issa Sabi, and the LIFE Study Consortium

**Affiliations:** ^1^ Mbeya Medical Research Center National Institute for Medical Research (NIMR) Mbeya Tanzania; ^2^ Institute of Infectious Diseases and Tropical Medicine LMU University Hospital LMU Munich Munich Germany; ^3^ Institute for Medical Information Processing Biometry and Epidemiology (IBE) Faculty of Medicine LMU Munich Pettenkofer School of Public Health Munich Germany; ^4^ Instituto Nacional de Saúde (INS) Maputo Mozambique; ^5^ David Geffen School of Medicine University of California Los Angeles California USA; ^6^ Clinton Health Access Initiative (CHAI) Maputo Mozambique; ^7^ Instituto Universitário Egas Moniz (IUEM) Lisbon Portugal; ^8^ German Center for Infection Research (DZIF) partner site Munich Munich Germany; ^9^ Fraunhofer Institute for Translational Medicine and Pharmacology ITMP Immunology, Infection and Pandemic Research Munich Germany; ^10^ Unit Global Health Helmholtz Center Munich German Research Center for Environmental Health (HMGU) Neuherberg Germany

**Keywords:** HIV acquisitions, infant, newborn, point‐of‐care systems, risk assessment, viral load

## Abstract

**Introduction:**

Despite global reductions in vertical HIV transmission (VHT), 120,000 children newly acquired HIV in 2023. High maternal viral load (VL) is a major risk factor for VHT. We estimated the impact of point‐of‐care (PoC) maternal VL testing at delivery in profiling the risk of VHT and its impact on appropriate postnatal prophylaxis for infants born to women living with HIV (WLWH).

**Methods:**

The cluster‐randomized LIFE (Long term Impact on inFant hEalth) study was conducted at 28 health facilities in Tanzania and Mozambique from 2019 to 2021. At delivery, the intervention arm applied PoC maternal VL plus clinical criteria for VHT risk assessment, while the control arm used clinical criteria only. In Tanzania, both arms provided ePNP based on maternal risk factors, while Mozambique provided ePNP universally. We used mixed effects logistic regression to estimate the intervention effect on the proportion of infants at high risk (Tanzania and Mozambique) and infants at high risk receiving ePNP (Tanzania only).

**Results:**

A total of 6467 WLWH were enrolled: 66.3% were diagnosed before the third trimester, 99% were on antiretroviral therapy and 78% were virally suppressed at delivery. Of 6564 newborns of WLWH included, 774 (11.7%) were identified to be at a high risk: 629 (19.3%) versus 145 (4.4%) in intervention and control arms, respectively; *p*<0.0001. In the intervention arm, 520 (82.7%) infants at high risk were classified only based on maternal PoC VL at delivery. In the control arm, 720 (21.8%) additional infants at high risk would have been identified if their mothers had received PoC VL assessment. In Tanzania, infants at high risk in the intervention arm were significantly more likely to receive ePNP: 59.5% versus 31.4% (OR 4.42, 95% CI: 1.09, 17.89). However, 40.5% from intervention arm and 68.6% from control arm did not receive ePNP despite high‐risk classification at delivery.

**Conclusions:**

PoC maternal VL testing at delivery significantly increased the proportion of infants identified to be at high risk. Infants at high risk whose mothers received PoC VL at delivery were more often initiated on ePNP. However, the linkage of infants at high risk to appropriate prophylaxis remains suboptimal, warranting consideration of universal ePNP.

## 1 INTRODUCTION

Improved access and availability of antiretroviral therapy (ART) has to date prevented up to 200,000 new HIV acquisitions among infants globally. Still, about 120,000 children aged 0–14 years acquired HIV globally in 2023, totalling the number of children living with HIV to 1.4 million [1]. In many African countries, including Tanzania and Mozambique, ART coverage among pregnant women is high: 94% in Tanzania in 2019 and 99% in Mozambique in 2022 [2, 3]. Nonetheless, both Tanzania and Mozambique rank among priority countries with high vertical HIV transmission (VHT), with rates of 6.5% for Tanzania and 10.4% for Mozambique through the breastfeeding period [2, 4].

Maternal viral load (VL) and duration on ART are the most critical determinants of VHT [5, 6, 7, 8, 9, 10]. The WHO defines infants at high risk as those whose mother was first identified as HIV positive at delivery or postpartum, acquired HIV during pregnancy or the breastfeeding period, started ART less than 4 weeks prior to delivery or did not achieve viral suppression (<1000 copies/ml) in the 4 weeks before delivery [8, 11]. The current Tanzanian guidelines recommend that mothers who are already on ART have their VL tested during their first antenatal care visit. If their VL is well‐controlled (below 50 copies/ml), they should have a follow‐up VL test every 6 months until they stop breastfeeding [12]. In Mozambique, the first VL is performed on entry to antenatal care if ART was started more than 3 months prior [13]. In both countries, pregnant women newly diagnosed with HIV or initiated on ART are recommended to have a VL test 3 months after ART initiation, followed by a repeat test every 6 months, ideally at 36–40 weeks’ gestation [12, 13]. However, this schedule of VL testing during pregnancy does not guarantee the availability of a VL result 4 weeks before or at the time of delivery.

Currently, infants at high risk for VHT should receive enhanced post‐natal prophylaxis (ePNP): zidovudine (AZT) plus nevirapine (NVP) or AZT plus lamivudine (3TC) plus NVP for the first 6 weeks of life, followed by an additional 6 weeks of NVP for breastfeeding infants [8, 11]. Infants without high‐risk criteria should receive prophylaxis with NVP for 6 weeks following birth. In Tanzania, ART prophylaxis follows the risk‐based approach described above [12]. However, the identification of mother‐infant pairs at high risk for VHT is still insufficient in many settings, due to a paucity of reliably recorded data, and infants born to mothers at high risk may not receive the appropriate postnatal HIV prophylaxis regimen [14]. Therefore, Mozambique provides ePNP to all infants born to women living with HIV (WLWH) regardless of high‐risk criteria [13].

Decentralized maternal VL monitoring using point‐of‐care (PoC) or near PoC systems during Antenatal Care (ANC) has been shown to increase the proportion of mothers receiving VL testing and decrease the turnaround time for results and clinical action for those with unsuppressed VL [15, 16, 17]. The availability of PoC VL tests at a health facility further ensures access to timely VL results at or around the time of delivery, information crucial for VHT risk assessment [18]. We assessed the impact of PoC maternal VL at delivery in determining the VHT risk in Mozambique and Tanzania and subsequent clinical decisions on postnatal prophylaxis (PNP) regimen for infants born to WLWH in Tanzania.

## 2 METHODS

### 2.1 Study design

The LIFE (Neonatal HIV early infant diagnosis [EID] versus standard of care EID—Long term Impact on inFant hEalth) study was a cluster‐randomized, controlled trial primarily evaluating the clinical impact of PoC test‐and‐treat procedures for infants and neonates at birth and week 4–6 versus standard‐of‐care PoC early infant diagnosis at 4–6 weeks only [19, 20]. This manuscript reports on a secondary objective of the trial to assess the impact of maternal PoC VL at the time of delivery in addition to clinical VHT criteria (intervention) compared to the standard‐of‐care of using clinical VHT criteria only (control) on VHT high‐risk detection and subsequent provision of standard infant PNP for low‐risk infants or ePNP for infants at high risk in Tanzania. We assessed these outcomes at delivery among mother‐child pairs enrolled in the LIFE study between October 2019 and September 2021. This study was conducted by the National Institute for Medical Research in Mbeya, Tanzania (NIMR‐Mbeya), the Instituto Nacional de Saúde (INS) in Mozambique, and sponsored by LMU University Hospital, LMU Munich in Germany.

### 2.2 Study settings and participants

The LIFE study was conducted at 28 maternity health centres in rural primary healthcare settings in Mozambique and Tanzania. Pregnant WLWH, aged 18 years and older, were included in the study until 72 hours post‐delivery. Mother‐infant pairs were excluded from the study in the case of stillbirth, if the infant or mother required emergency medical care or if the mother had deficiencies rendering it difficult to take part in the study or understand study information.

### 2.3 Study procedures

For VHT high‐risk identification, half of the facilities implemented the intervention using clinical screening plus maternal PoC VL testing at delivery, and the other half used clinical and ANC information only. To assess VL also for mothers in the control group, maternal blood samples obtained at the time of delivery were sent to referral laboratories for retrospective VL testing, and results were communicated once available, usually in the subsequent visits. Retrospective VL results in the control group had no impact on PNP decisions. Maternal PoC VL testing at the intervention sites was performed using the Cepheid Xpert® HIV‐1 VL (Cepheid, Sunnyvale, USA) in Tanzania and the Abbott mPIMA™ HIV‐1/2 VL (Abbott, Chicago, USA) in Mozambique. PoC platforms were placed in an accessible room close to the delivery unit, and nurses and laboratory personnel at the facilities were trained to run the tests. Central laboratory VL testing was performed using HIV‐1/2 Roche TaqMan (Roche Diagnostics, Branchburg, USA) or the GeneXpert platform. EDTA blood samples were centrifuged at the sites. For the Abbott mPIMA system used in Mozambique, 50 µl of plasma was transferred into the testing cartridge, and the results were received after a turnaround time of approximately 60 minutes. The mPIMA VL test has a threshold for detection of 800 copies/ml. For the GeneXpert system used in Tanzania, 1 ml of plasma was transferred into the testing cartridge, and the results were received after a turnaround time of 90 minutes. The GeneXpert test has a threshold for detection of 40 copies/ml.

Clinical assessment of VHT risk among mothers was performed using WHO‐defined clinical criteria. In the context of this study, this included: (1) mother not on ART or on ART <4 weeks before delivery; (2) known VL ≥1000 copies/ml during past 4 weeks before delivery; (3) newly HIV diagnosed at delivery, or incident HIV acquisitions during pregnancy (defined as new HIV diagnosis in woman with a prior negative HIV test during pregnancy); and (4) significant obstetric complications as judged by the obstetrician. PoC VL results ≥1000 copies/ml at delivery were considered high risk for VHT. All mothers with high‐risk criteria were offered enhanced adherence counselling to identify and address ART adherence barriers according to routine procedures.

All neonates born to mothers living with HIV in the intervention arm received PoC EID testing at birth and at week 4–6, whereas neonates from the control group received a first PoC EID at week 4–6, according to Standard of Care (SoC) procedures [20]. All confirmed HIV‐positive neonates or infants were offered immediate ART. Infants diagnosed with HIV at birth (*n* = 38) were excluded from analysis. All neonates with a negative PoC HIV test at birth in the intervention group and all neonates in the control group received post‐natal prophylaxis: in Tanzania, standard NVP prophylaxis for 6 weeks for neonates considered low‐risk (PNP) or AZT plus 3TC plus NVP for the first 6 weeks followed by NVP only until week 12 if considered at high risk for VHT (ePNP) and, in Mozambique, ePNP irrespective of VHT risk criteria according to national guidelines [12, 13].

### 2.4 Outcomes and statistical analysis

We evaluated the proportion of infants born to WLWH at birth with clinical high‐risk criteria for VHT in Mozambique and Tanzania and estimated the additional impact of PoC VL screening at delivery to improve high‐risk identification. The impact of PoC VL results available at delivery on the PNP regimen provided was assessed by comparing the infant's VHT risk status with the PNP regimen received for Tanzania only, as Mozambique provided universal ePNP irrespective of VHT risk. Infants diagnosed HIV positive at birth in the intervention group were excluded from the analysis.

Qualitative EID PoC test results were binary coded as positive or negative. The VL cutoff for meeting high‐risk criteria for mothers was 1000 copies/ml. Categorical data was summarized using counts and percentages, and continuous data was summarized using the mean and standard deviation (SD) or median and range. Given the cluster‐randomized design and binary outcome, we used mixed effects logistic regression models to estimate the effect of the intervention on the proportion of infants identified at high risk (for both countries) and on infants at high risk receiving ePNP (for Tanzania only) [21]. We clustered the standard errors to account for randomization at the health facility level and multiple births. Statistical tests were performed assuming a two‐sided hypothesis, and *p*‐values less than 0.05 were considered sufficient statistical evidence to reject the null hypothesis.

### 2.5 Ethical considerations

Informed consent was obtained from all participants before study inclusion, and participants were assured confidentiality and freedom of participation in the study. Ethical clearance was granted by the Comité Institucional de Bioética para a Saúde do INS (CIBS‐INS) in Mozambique, Mbeya Medical Research and Ethics Committee (MMREC), the National Health Research Ethics Review Committee in Tanzania, and the ethics committee of the Medical Faculty of the University of Munich (LMU). This study was registered with Clinicatrials.gov (Ref NCT04032522).

## 3 RESULTS

A total of 6467 mothers living with HIV and their 6564 newborns born to WLWH, enrolled in the study between October 2019 and September 2021, were included in this analysis. Mother's mean age was 29.5 (SD 5.84) years, and the majority (85.1%) had completed at least primary school education. In the intervention arm, 94.3% of mothers reported that they had disclosed their HIV status to their partner or another trusted person, compared to 92.4% in the control arm. Median time on ART was 5.8 and 6.5 months in the intervention and control arms, respectively. Most mothers were on a DTG‐containing ART regimen at delivery (80.6% and 72.4% in the intervention and control arms, respectively). Antenatal care attendance by the second trimester was 93.5% in the intervention arm, compared to 87.7% in the control arm. Most mothers were virally suppressed at delivery, 79.5% and 73.2% in the intervention and control arms, respectively, with higher proportions of suppressed VL in Tanzania compared to Mozambique (Table 1).

**Table 1:** Demographic characteristics and baseline measurements of included mothers living with HIV

	Mozambique		Tanzania		Overall	
	Intervention (*N* = 2032)	Control (*N* = 1906)	Intervention (*N* = 1179)	Control (*N* = 1350)	Intervention (*N* = 3211)	Control (*N* = 3256)
**Age** Mean (SD)	29.0 (5.48)	28.6 (5.43)	30.6 (6.28)	30.4 (6.24)	29.6 (5.84)	29.4 (5.84)
**Education** *N* (%)						
None	279 (13.7%)	252 (13.2%)	202 (17.1%)	229 (17.0%)	481 (14.9%)	481 (14.8%)
Primary school	776 (38.2%)	773 (40.6%)	811 (68.8%)	893 (66.1%)	1587 (49.4%)	1666 (51.2%)
Secondary school or above	977 (48.1%)	881 (46.2%)	166 (14.1%)	228 (16.9%)	1143 (35.6%)	1109 (34.1%)
**HIV diagnosis** *N* (%)						
Before third trimester	1401 (68.9%)	1291 (67.7%)	709 (60.1%)	885 (65.6%)	2110 (65.7%)	2176 (66.8%)
Third trimester or later	624 (30.7%)	596 (31.3%)	264 (22.4%)	311 (23.0%)	888 (27.7%)	907 (27.9%)
Unknown	7 (0.3%)	19 (1.0%)	206 (17.5%)	154 (11.4%)	213 (6.6%)	173 (5.3%)
**Self‐reported HIV disclosure** *N* (%)	1926 (94.8%)	1729 (90.7%)	1101 (93.4%)	1279 (94.7%)	3027 (94.3%)	3008 (92.4%)
**ART regimen** *N* (%)						
TDF + 3TC + DTG	1719 (84.6%)	1523 (79.9%)	870 (73.8%)	835 (61.9%)	2589 (80.6%)	2358 (72.4%)
TDF + 3TC + EFV	280 (13.8%)	358 (18.8%)	291 (24.7%)	500 (37.0%)	571 (17.8%)	858 (26.4%)
Other	2 (0.1%)	3 (0.2%)	3 (0.3%)	4 (0.3%)	5 (0.2%)	7 (0.2%)
None	31 (1.5%)	22 (1.2%)	15 (1.3%)	11 (0.8%)	46 (1.4%)	33 (1.0%)
**Time on ART (months)** Median [Min, Max]	5.06 [0, 164]	5.26 [0, 221]	8.29 [0, 278]	12.4 [0, 282]	5.77 [0, 278]	6.48 [0, 282]
**ANC attendance by second trimester** *N* (%)	1884 (92.7%)	1621 (85.0%)	1119 (94.9%)	1234 (91.4%)	3003 (93.5%)	2855 (87.7%)
**Gravida** Median [Min, Max]	3.00 [1.00, 12.0]	3.00 [1.00, 12.0]	3.00 [1.00, 10.0]	3.00 [1.00, 10.0]	3.00 [1.00, 12.0]	3.00 [1.00, 12.0]
**Para** Median [Min, Max]	2.00 [0, 10.0]	2.00 [0, 11.0]	2.00 [0, 9.00]	2.00 [0, 9.00]	2.00 [0, 10.0]	2.00 [0, 11.0]
**Delivery mode** *N* (%)						
Vaginal	2031 (100.0%)	1906 (100%)	995 (84.4%)	1188 (88.0%)	3026 (94.2%)	3094 (95.0%)
Caesarean	1 (0.0%)	0 (0%)	184 (15.6%)	162 (12.0%)	185 (5.8%)	162 (5.0%)
**HIV plasma viral load at delivery** ^a^ *N* (%)						
<1000 copies/ml	1494 (73.5%)	1209 (63.4%)	1060 (89.9%)	1174 (87.0%)	2554 (79.5%)	2383 (73.2%)
≥1000 copies/ml	531 (26.1%)	661 (34.7%)	103 (8.7%)	96 (7.1%)	634 (19.7%)	757 (23.2%)
Not available	7 (0.3%)	36 (1.9%)	16 (1.4%)	80 (5.9%)	23 (0.7%)	116 (3.6%)

Abbreviations: ANC, antenatal care; ART, antiretroviral treatment; DTG, dolutegravir; EFV, efavirenz; TDF, tenofovir; 3TC, lamivudine.

^a^ In the intervention arm, PoC VL was performed at the site and immediately available during the delivery encounter; in the control arm, VL was measured at the centralized laboratory with results available at a subsequent visit. Gravida—total number of confirmed pregnancies a female has had. Para—the number of births that a female has had.

**Figure 1:**
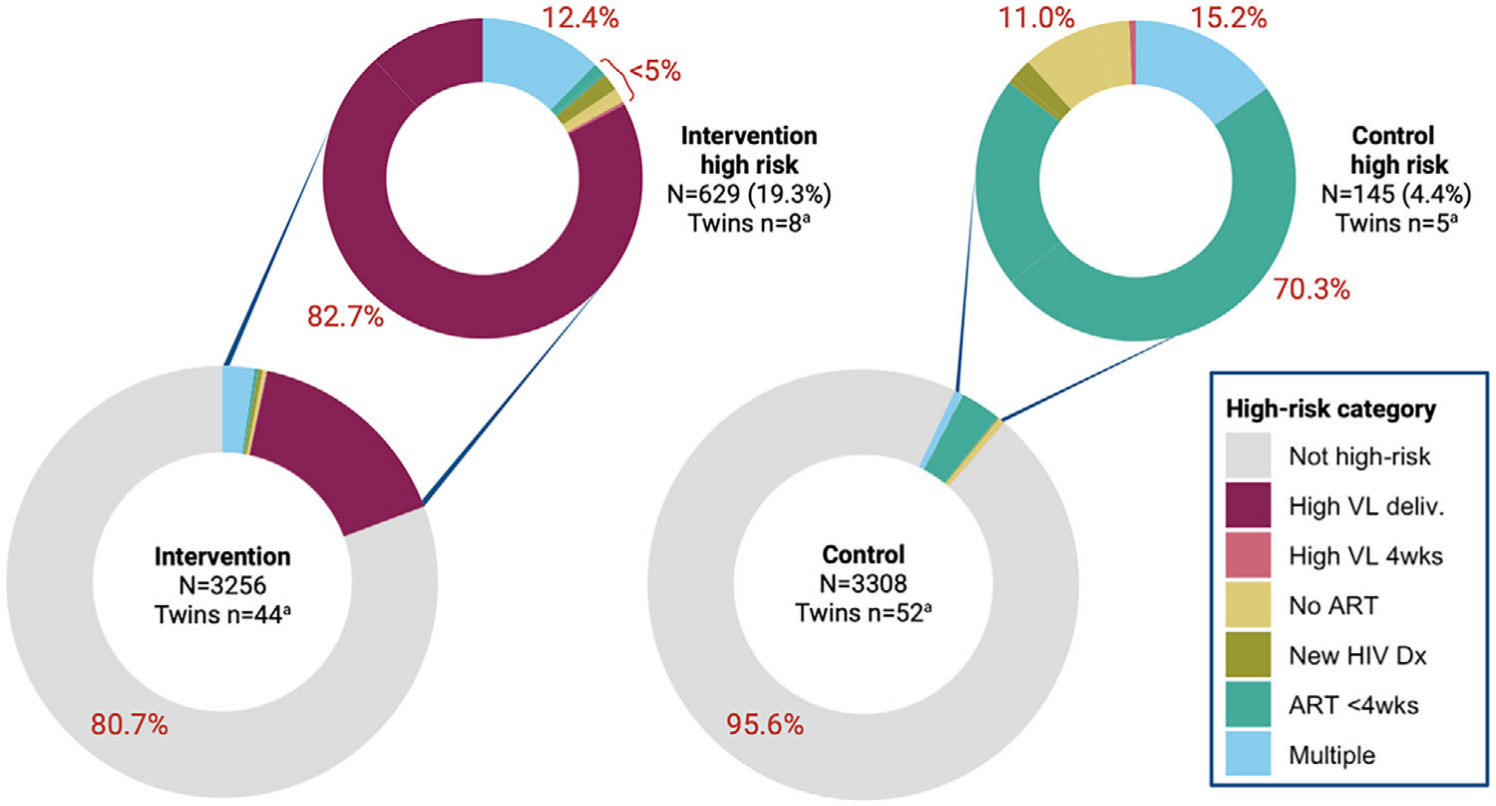
Proportions of infants at high risk for vertical HIV transmission by study arm. Denominator includes all included infants (large donuts) and all infants at high risk (small donuts). ^a^Number of twins/multiples included in denominator. ART <4wks, initiated antiretroviral treatment less than 4 weeks prior to delivery; High VL deliv., high viral load at delivery; High VL 4wks, high viral load in the past 4 weeks; Multiple, multiple high‐risk criteria; New HIV Dx, newly diagnosed with HIV at delivery.

Using information available to health providers at the time of delivery, 629 (19.3%) infants from the intervention arm and 145 (4.4%) from the control arm were identified to be at high risk for VHT (OR 4.81, 95% confidence interval [CI]: 2.89, 8.00; *p*<0.0001; Figure 1 and Table 2). Eight high risk twins were in the intervention arm and five high risk twins were in the control arm. In the intervention arm, 82.7% (520) of infants at high risk were identified to be at high risk by maternal PoC VL alone. An additional 12.2% (77) had mothers with a VL ≥1000 copies/ml plus one or more clinical high‐risk criteria; only one mother with multiple criteria for high‐risk status had a VL <1000 copies/ml. Only 24 (0.75%) mothers in the intervention arm did not have VL results available at delivery, primarily due to a lack of test kits available at the health facility. In the control arm, infants at high risk were more commonly identified because their mothers were either on ART <4 weeks (70.3%), not on ART (11.0%) or had multiple clinical high‐risk criteria (15.2%). VL results from the prior 4 weeks were rarely available in either study arm. There were no differences in high‐risk status by infant sex (*p* = 0.755).

**Table 2:** Frequency and proportion of infants at high risk for vertical HIV transmission

	Mozambique		Tanzania		Overall	
	Intervention (*N* = 2057)	Control (*N* = 1926)	Intervention (*N* = 1199)	Control (*N* = 1382)	Intervention (*N* = 3256)	Control (*N* = 3308)
**High‐risk category**						
Not high risk	1539 (74.8%)	1832 (95.1%)	1088 (90.7%)	1331 (96.3%)	2627 (80.7%)	3163 (95.6%)
High VL at delivery^a^	446 (21.7%)	n/a	74 (6.2%)	n/a	520 (16.0%)	n/a
High VL 4 weeks	2 (0.1%)	0 (0%)	0 (0%)	1 (0.1%)	2 (0.1%)	1 (0.0%)
No ART	6 (0.3%)	11 (0.6%)	3 (0.3%)	5 (0.4%)	9 (0.3%)	16 (0.5%)
New HIV diagnosis	0 (0%)	1 (0.1%)	11 (0.9%)	3 (0.2%)	11 (0.3%)	4 (0.1%)
ART <4 weeks	7 (0.3%)	71 (3.7%)	2 (0.2%)	31 (2.2%)	9 (0.3%)	102 (3.1%)
Multiple criteria^b^	57 (2.8%)	11 (0.6%)	21 (1.8%)	11 (0.8%)	78 (2.4%)	22 (0.7%)
**Any high‐risk criteria**	518 (25.2%)	94 (4.9%)	111 (9.3%)	51 (3.7%)	629 (19.3%)	145 (4.4%)

Abbreviations: ART, antiretroviral treatment; VL, viral load.

^a^ In the intervention arm, PoC VL was performed at the site and immediately available during the delivery encounter; in the control arm, VL was measured at the centralized laboratory with results available at a subsequent visit.

^b^ Two or more criteria for high‐risk status. Only one mother in the intervention arm in Mozambique did not have VL ≥1000 copies/ml at delivery as one of the criteria for high‐risk status.

**Figure 2:**
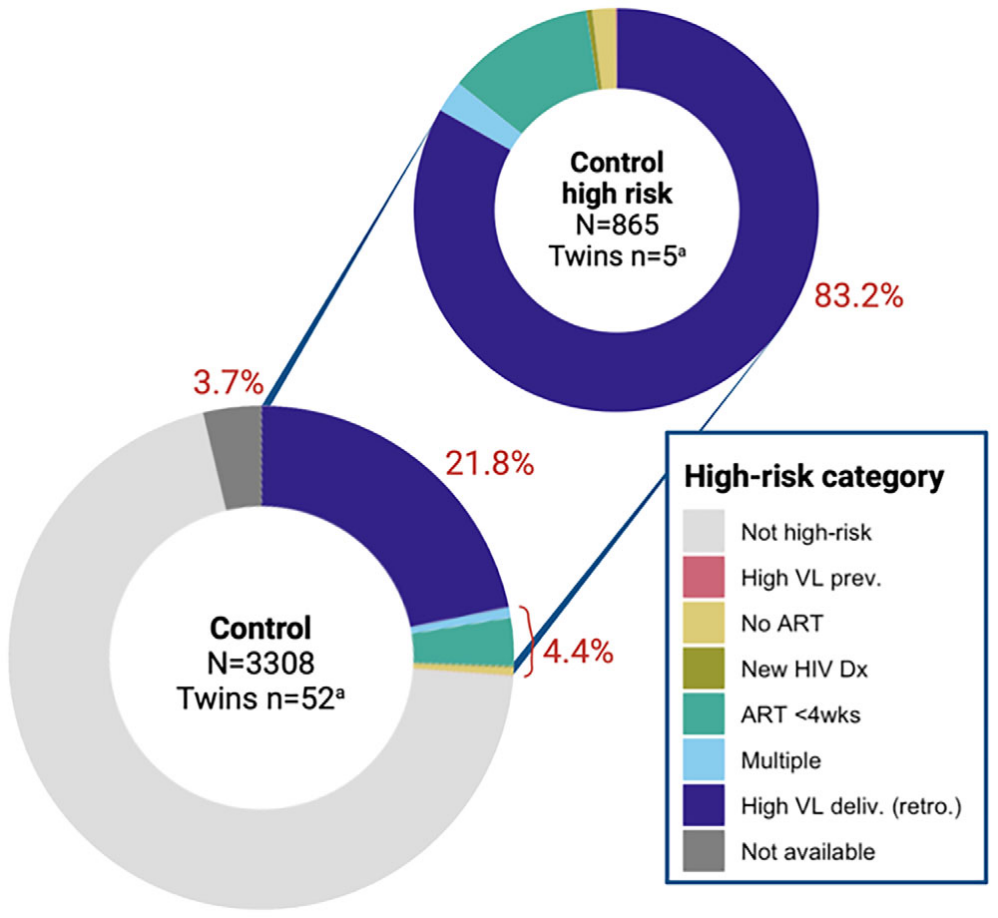
Proportion of infants at high risk for vertical HIV transmission in the control arm including retrospective VL results at delivery, representing the proportion of infants who could have been identified as high risk if maternal PoC VL testing had been available at the health facility (dark blue). Reasons for high‐risk classification indicated by colours. Multiple high‐risk category includes two or more reasons for high‐risk classification. Denominator includes all included infants. High VL deliv. (retro) refers to VL testing performed retrospectively at the central laboratory and not available in real‐time. NA represents infants for whom retrospective maternal VL results were not available due to sample lost in transit to central laboratory, coagulated blood or otherwise unusable sample, or result not recorded or recorded as “error.” ^a^Number of twins/multiples included in denominator. ART <4wks, initiated antiretroviral treatment less than 4 weeks prior to delivery; High VL deliv. (retro.), high viral load at delivery, measured retrospectively; High VL 4wks, high viral load in the past 4 weeks; Multiple, multiple high‐risk criteria; New HIV Dx, newly diagnosed with HIV at delivery.

Based on retrospective VL testing performed at the central laboratory, an additional 21.8% (720) of infants born to mothers living with HIV from the control arm who were not already identified to be at high risk had mothers with unsuppressed VL ≥1000 copies/ml at delivery (Figure 2). These infants would have represented 83.2% of infants at high risk in the control arm who could be identified to be at high risk solely due to maternal VL at delivery, similar to the proportion seen in the intervention arm. An additional 3.7% (125) of mothers did not have retrospective VL results available due to sample clotting or loss of samples, or results between health facilities and the central labs. Sample or result loss was notably higher when performed at the central lab compared to at PoC.

In the LIFE study, nurses identified infants at high risk if any of the above criteria were met. However, as maternal VL is the most direct indicator for VHT risk, suppressed VL may overrule other risk factors. Thus, 4.0% (25) of infants at high risk in the intervention arm would have been downgraded to low risk. In the control arm, 69.9% (100) of infants at high risk would have been downgraded to low risk, primarily due to mothers who had been on ART <4 weeks but were suppressed at delivery (Table S1). Therefore, 18.5% (604) and 23.1% (765) of infants would be considered to be at high risk for VHT in the intervention and control arms, respectively. Further, 98.8% and (at least) 94.1% of these infants could be identified as being at high risk by maternal VL alone.

A total of 81 infants were first diagnosed HIV positive between 4 and 16 weeks of age: 31 in the intervention arm and 50 in the control arm. Twenty‐seven infants in the control arm were retrospectively identified as positive at birth, including six who died or were lost to follow‐up after birth and were, therefore, never diagnosed. These 27 infants would have benefited from birth testing and immediate ART initiation. Among the 31 infants from the intervention arm, 77.4% (24/31) had mothers with VL ≥1000 copies/ml at delivery, 6.5% (2/31) had multiple high‐risk criteria including VL ≥1000 copies/ml at delivery, one's mother was virally suppressed but did not have ART documented, and 12.9% (4/31) did not have any high‐risk criteria. In the control arm, only 14.0% (7/50) of positive infants, including those who already acquired HIV at birth, were identified to be at high risk based on clinical information available at delivery (Table S2). However, an additional 88.4% (38/43) would have been classified as being at a high risk based on retrospective maternal VL results.

**Figure 3:**
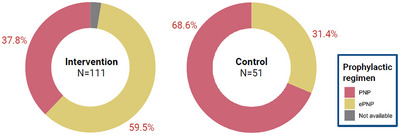
Proportion of infants at high risk in Tanzania initiated on enhanced postnatal HIV prophylaxis. Colours indicate prophylactic regimen. ePNP, enhanced postnatal prophylaxis; PNP, postnatal prophylaxis.

In Tanzania, infants at high risk in the intervention arm had 4.42 (95% CI: 1.09, 17.89) times the odds of being initiated on ePNP compared to those in the control arm after adjusting for calendar time, health facility and twins (*p* = 0.037) (Table 3). Overall, only 50.6% of infants at high risk were offered ePNP: 59.5% of infants at high risk in the intervention arm, despite the availability of PoC VL results at delivery and correct identification of high‐risk status, compared to 31.4% in the control arm (Figure 3), with no differences observed by infant sex (*p* = 0.252). Of the nine infants who acquired HIV between 4 and 16 weeks of age in Tanzania, only three received ePNP.

**Table 3:** In Tanzania only, proportions of infants receiving regular (PNP) versus enhanced (ePNP) prophylaxis by study arms

	Intervention		Control		
	Low risk (*N* = 1088)	High risk (*N* = 111)	Low risk (*N* = 1331)	High risk (*N* = 51)	aOR (95% CI)^a^
**Prophylactic regimen** *N* (%)				
PNP	1054 (96.9%)	42 (37.8%)	1316 (98.9%)	35 (68.6%)	ref
ePNP	13 (1.2%)	66 (59.5%)	12 (0.9%)	16 (31.4%)	4.424 (1.094, 17.89)
None/unknown	21 (1.9%)	3 (2.7%)	3 (0.2%)	0 (0%)	−

Abbreviations: aOR, adjusted odds ratio; CI, confidence interval; ePNP, enhanced postnatal prophylaxis; PNP, postnatal prophylaxis.

^a^ Mixed effects model with fixed effects for study arm and calendar time and random effects for health facility and multiple birth.

## 4 DISCUSSION

This study demonstrated that PoC maternal VL testing at delivery in rural and semi‐urban primary healthcare settings identifies more mother‐infant pairs at high risk for VHT and results in more infants at high risk receiving the recommended enhanced ART PNP regimen in Tanzania. However, a gap remains in translating high‐risk identification into clinical action, as only 50.6% of infants at high risk received ePNP in Tanzania, even with PoC maternal VL results available at delivery.

The availability of VL results at delivery significantly improved the ability of healthcare workers to identify mother‐infant pairs at high risk. Facilities where PoC was available for maternal VL testing at delivery were more than four times as likely to identify newborns at high risk for VHT as compared to those that relied on clinical criteria only. Suboptimal monitoring of maternal VL during pregnancy and delivery poses a significant challenge for healthcare workers to identify infants at high risk and intervene in a timely manner [22, 23, 24]. Many countries, including Mozambique and Tanzania, base the timing of VL testing among pregnant women with HIV on the time since ART initiation. A simulation study using data from South Africa showed that 69% of pregnant women would not receive a VL test at all during pregnancy under this scenario [25], let alone in the 4 weeks prior to delivery. Thus, VL testing schedules for pregnant women may be more appropriate if based on gestational age rather than time since ART initiation, with a single VL test at 36 weeks’ gestation best at predicting high VL at delivery. However, considering that mobility from one facility to another for delivery is common in sub‐Saharan Africa, turnaround times are potentially long and documentation of results is often lacking [14], it may be impractical to expect that records will be available at the time of delivery for clinical decision‐making. As shown in Zimbabwe, PoC VL monitoring during ANC resulted in higher uptake of VL monitoring, prompt availability of results and more frequent clinical action for those with unsuppressed VL [16]. While ANC attendance was high in our study, PoC or near PoC VL monitoring at delivery would still identify more mother‐infant pairs in need of further monitoring and intervention, and reach mothers who may more often be at high risk because they do not attend ANC. Only 0.75% of mothers in the intervention arm did not have the same‐day VL results due to unavailability of testing kits, notably lower than the proportion of unavailable VLs performed at the central laboratory in the control arm (3.7%). Still, establishing contingency plans such as alternative referral pathways and supply chain strengthening are necessary to ensure continuity of these diagnostic services, especially in limited resource settings.

Newborns in the intervention arm at high risk for VHT were more likely to be initiated on the appropriate (ePNP) regimen for ART prophylaxis compared to the control arm. Timely availability of maternal VL results may have motivated healthcare workers to initiate neonates at high risk on ePNP since viraemia is a well‐known risk factor for HIV transmission. However, approximately half of all newborns at high risk were not initiated on an enhanced prophylaxis, including six who subsequently acquired HIV during the post‐natal prophylaxis period. This indicates poor uptake of appropriate care and treatment measures for newborns at high risk among healthcare workers, or less likely, stock‐outs of appropriate antiretrovirals, as the study ensured these were available. Similar to our results, a study in Zimbabwe found that the uptake of ePNP was poor, with the majority of newborns being initiated on monotherapy prophylaxis despite being classified as being at high risk [14]. The recommendation for risk‐based ePNP provision was introduced in Tanzania [12] around the same time as study initiation, and, therefore, our results may reflect a lag in implementation, which could have improved beyond the study period. This does not discount the importance of assessing other barriers from high‐risk identification to clinical action at patient, healthcare worker, health facility and structural levels. Further studies are needed to dissect the reasons for the gap in clinical action following high‐risk classification to inform mitigation strategies.

The use of enhanced PNP has been shown to prevent VHT and has been widely recommended for infants at higher risk of transmission [26, 27, 28]. Several combinations ranging from dual to triple ART for varying lengths of time have been recommended. In our study, most infants who acquired HIV in the post‐partum period were from Mozambique, where ePNP was provided universally. The discussion of whether countries like Tanzania, which have relatively low VHT rates, should opt for universal ePNP should be carefully considered. Our study is limited in evaluating the impact of ePNP provision based on risk criteria, as applied in Tanzania, on VHT due to very low transmission rates. Adverse events, including anaemia and neutropenia, have been reported with the use of combination ART for neonatal prophylaxis [27, 29, 30], more commonly in the early months of life, although most studies agree these regimens are tolerable [26, 29, 30]. Still, the universal use of ePNP would necessitate the availability of monitoring for haematological parameters for infants born to WLWHW, which may be scarce in many limited resource settings.

This study had some limitations: first, there was no a priori sample size calculation for this analysis as the outcome was a secondary objective for the LIFE study. Thus, the numbers used were adopted from the main study sample size and assumptions. Second, the potential for residual confounding in the relationship between PoC VL and ePNP initiation, especially factors related to healthcare workers’ individual characteristics affecting their care delivery and facility‐level infrastructure, including ePNP supply, remains a concern. However, we are confident that the site selection in the LIFE study balanced facility‐level characteristics well to maximize generalizability to similar settings.

## 5 CONCLUSIONS

The findings from this study underscore the significant impact of PoC maternal VL testing at delivery in augmenting the capacity of healthcare workers to accurately identify mother‐infant pairs at high risk for VHT. Having timely and precise data at their disposal, healthcare providers can make informed decisions about VHT risk, including initiating newborns on the enhanced post‐natal prophylaxis as appropriate. However, the linkage of infants at high risk to appropriate PNP remains suboptimal in Tanzania. Thus, health systems and decision‐makers should allocate resources to strengthen the ability of healthcare workers to translate the identification of infants at high risk into clinical action or consider a universal ePNP approach.

## COMPETING INTERESTS

We declare that none of the authors have any competing interests that have influenced the conduct of the study and write‐up of this manuscript.

## AUTHORS’ CONTRIBUTIONS

IS, AK, IJ, NT, NEN and MH acquired the funds and designed the study. SB, RE, AM, BM, WCB, JL, KP, NC, FC, CM and MM contributed to overall study conduct and data collection. KE and AFL idealized and performed data analysis. AFL and KE drafted the manuscript. All coauthors had full access to the data, reviewed the final version of the manuscript and accepted the responsibility to submit for publication.

## FUNDING

This study was supported by the European and Developing Countries Clinical Trials Partnership (EDCTP, RIA2016MC‐1615), UNITAID (UCPOC2B) and the German Center for Infection Research (DZIF, TTU 04.708).

## Supporting information




**Table S1**: Frequency and proportion of reasons for vertical HIV transmission high‐risk classification and Viral Load (VL) results performed at the point‐of‐care (intervention) versus retrospectively at the central laboratory (control). Infants who could be downgraded to low‐risk status based on VL results alone are indicated in yellow, infants categorized as high‐risk with maternal VL ≥1000 copies/ml at delivery are shown in green.
**Table S2**: Frequency and proportion of HIV‐positive infants with high‐risk criteria for vertical HIV transmission between week 4 and 16 of life by study arm and country according to information available to health providers at delivery.

## Data Availability

Individual participant data that underlie the results presented in this article (text, tables, figures and supplemental material) with labels defining all fields will be made available upon publication to researchers whose proposed use of the data has been approved by the study steering committee. Proposals should be directed to akroidl@lrz.uni-muenchen.de. Researchers wishing to access this data will be asked to sign a data use agreement.

## References

[1] UNAIDS . The urgency of now: AIDS at a crossroads. Jt United Nations Program HIV/AIDS. 2024;150. Available from: https://www.unaids.org/sites/default/files/media_asset/2024‐unaids‐global‐aids‐update_en.pdf

[2] da Saúde M . Relatório Anual 2022. 2023.

[3] UNICEF . The mother of all prevention. UNICEF United Republic of Tanzania [Internet]. [cited 2022 Sep 21]. Available from: https://www.unicef.org/tanzania/stories/mother‐all‐prevention

[4] PMTCT annual report of Tanzania. 2023.

[5] Bardeskar NS , Ahir‐Bist SP , Mehta PR , Samant‐Mavani P , Nanavati R , Mania‐Pramanik J . Anti‐retroviral therapy failure in HIV‐1 infected pregnant women and its associated risk of HIV transmission. Arch Gynecol Obstet. 2020;302(5):1229–1235. 32803392 10.1007/s00404-020-05743-8

[6] Liu F , Liu GLZ . Factors responsible for mother to child transmission (MTCT) of HIV‐1 – a review. Eur Rev Med Pharmacol Sci. 2017;21:74–78. 29165760

[7] USAID . Prevention of Mother to Child Transmission (PMTCT). U.S. Agency for International Development. [cited 2022 Sep 8]. Available from: https://www.usaid.gov/global‐health/health‐areas/hiv‐and‐aids/technical‐areas/pmtct

[8] WHO . Consolidated guidelines on HIV prevention, testing, treatment, service delivery and monitoring: recommendations for a public health approach. Geneva: World Health Organization; 2021. 34370423

[9] Mock PA , Shaffer N , Bhadrakom C , Siriwasin W , Chotpitayasunondh T , Chearskul S , et al. Maternal viral load and timing of mother‐to‐child HIV transmission, Bangkok, Thailand. Bangkok Collaborative Perinatal HIV Transmission Study Group. AIDS. 1999;13(3):407–414. 10199232 10.1097/00002030-199902250-00014

[10] Moyo F , Haeri Mazanderani A , Murray T , Technau KG , Carmona S , Kufa T , et al. Characterizing viral load burden among HIV‐infected women around the time of delivery: findings from four tertiary obstetric units in Gauteng, South Africa. J Acquir Immune Defic Syndr. 2020;83(4):390–396. 31914002 10.1097/QAI.0000000000002267

[11] WHO . Consolidated guidelines on the use of antiretroviral drugs for treating and preventing HIV infection. 2016. 27466667

[12] Ministry of Health of Tanzania . The national guidelines for management of HIV and AIDS [Internet]. Dar es salaam; 2019. Available from: https://nacp.go.tz/download/national‐guidelines‐for‐the‐management‐of‐hiv‐and‐aids‐april‐2019/

[13] da Saúde M . Guião de cuidados do HIV do Adulto, Adolescente Grávida, Lactante e Criança. 2023.

[14] Komtenza B , Satyanarayana S , Takarinda KC , Mukungunugwa SH , Mugurungi O , Chonzi P , et al. Identifying high or low risk of mother to child transmission of HIV: how Harare City, Zimbabwe is doing? PLoS One. 2019;14(3):e0212848. 30865646 10.1371/journal.pone.0212848 PMC6415877

[15] Sacks JA , Fong Y , Gonzalez MP , Andreotti M , Baliga S , Garrett N , et al. Performance of Cepheid Xpert HIV‐1 viral load plasma assay to accurately detect treatment failure. AIDS. 2019;33(12):1881–1889. 31274537 10.1097/QAD.0000000000002303 PMC7024604

[16] Joseph J , Boeke CE , Makadzange EE , Sithole K , Maparo T , Mangwendeza PM , et al. Near‐point‐of‐care viral load testing during pregnancy and viremia at delivery. AIDS. 2022;36(5):711–9. 35025819 10.1097/QAD.0000000000003173

[17] Kufa T , Mazanderani AH , Sherman GG , Elie Mukendi A , Murray T , Moyo F , et al. Point‐of‐care HIV maternal viral load and early infant diagnosis testing around time of delivery at tertiary obstetric units in South Africa: a prospective study of coverage, results return and turn‐around times. J Int AIDS Soc. 2020;23(4):e25487. 32329186 10.1002/jia2.25487 PMC7180267

[18] Mashamba‐Thompson TP , Morgan RL , Sartorius B , Dennis B , Drain PK , Thabane L. Effect of point‐of‐care diagnostics on maternal outcomes in human immunodeficiency virus‐infected women: systematic review and meta‐analysis. Point Care. 2017;16(2):67–77. 29242711 PMC5726275

[19] Jani IV , Sabi I , Elsbernd K , Meggi B , Mahumane A , Lwilla AF , et al. Impact of point‐of‐care birth test‐and‐treat on clinical outcomes among infants with HIV: a cluster randomized trial in Mozambique and Tanzania. Clin Infect Dis. 2024;80(5):1114–1124. 10.1093/cid/ciae530 PMC12135909 39514367

[20] Kroidl A , Elsbernd K , Meggi B , Mahumane A , Lwilla AF , Boniface S , et al. Birth point‐of‐care test and treat reduces early mortality among HIV infected infants—CROI Conference [Internet]. [cited 2024 Apr 13]. Available from: https://www.croiconference.org/abstract/birth‐point‐of‐care‐test‐treat‐reduces‐early‐mortality‐among‐hiv‐infected‐infants/

[21] Hemming K , Taljaard M. Key considerations for designing, conducting and analysing a cluster randomized trial. Int J Epidemiol. 2023;52(5):1648–1658. 37203433 10.1093/ije/dyad064 PMC10555937

[22] Richardson TR , Esterhuizen TM , Engelbrecht AL , Slogrove AL. Recognition of infants at high risk for vertical HIV transmission at delivery in rural Western Cape Province, South Africa. S Afr Med J. 2022;112(11):860–865. 36420722 10.7196/SAMJ.2022.v112i11.16541

[23] Kafack EVF , Fokam J , Nana TN , Saniotis A , Halle‐Ekane GE. Evaluation of plasma viral‐load monitoring and the prevention of mother‐to‐child transmission of HIV‐1 in three health facilities of the Littoral region of Cameroon. PLoS One. 2022;17(11 November):1–12. 10.1371/journal.pone.0277271 PMC9639847 36342923

[24] Moyo F , Mazanderani AH , Kufa T , Sherman GG. Maternal HIV viral load testing during pregnancy and postpartum care in Gauteng Province, South Africa. South African Med J. 2021;111(5):469–473. 10.7196/SAMJ.2021.v111i5.15240 34852890

[25] Lesosky M , Glass T , Mukonda E , Hsiao NY , Abrams EJ , Myer L. Optimal timing of viral load monitoring during pregnancy to predict viraemia at delivery in HIV‐infected women initiating ART in South Africa: a simulation study. J Int AIDS Soc. 2017;20(Suppl 7):26–31. 10.1002/jia2.25000 PMC5978661 29171179

[26] Lallemant M , Amzal B , Sripan P , Urien S , Cressey TR , Ngo‐Giang‐Huong N , et al. Perinatal antiretroviral intensification to prevent intrapartum HIV transmission when antenatal antiretroviral therapy is initiated less than 8 weeks before delivery. J Acquir Immune Defic Syndr. 2020;84(3):313–322. 32205720 10.1097/QAI.0000000000002350 PMC9741956

[27] Nielsen‐Saines K , Watts DH , Veloso VG , Bryson YJ , Joao EC , Pilotto JH , et al. Three postpartum antiretroviral regimens to prevent intrapartum HIV infection. N Engl J Med. 2012;366:2368–2379. 22716975 10.1056/NEJMoa1108275 PMC3590113

[28] Cardenas MC , Farnan S , Hamel BL , Mejia Plazas MC , Sintim‐Aboagye E , Littlefield DR , et al. Prevention of the vertical transmission of HIV; a recap of the journey so far. Viruses. 2023;15(4):849. 37112830 10.3390/v15040849 PMC10142818

[29] Kakkar FW , Samson L , Vaudry W , Brophy J , Le Meur JB , Lapointe N , et al. Safety of combination antiretroviral prophylaxis in high‐risk HIV‐exposed newborns: a retrospective review of the Canadian experience. J Int AIDS Soc. 2016;19(1):20520. 26880241 10.7448/IAS.19.1.20520 PMC4753845

[30] Anugulruengkitt S , Suntarattiwong P , Ounchanum P , Srirompotong U , Jantarabenjakul W , Sophonphan J , et al. Safety of 6‐week neonatal triple‐combination antiretroviral postexposure prophylaxis in high‐risk HIV‐exposed infants. Pediatr Infect Dis J. 2019;38(10):1045–1050. 31365477 10.1097/INF.0000000000002426

