# Supplementary material for: Impact of point‐of‐care maternal viral load testing at delivery on vertical HIV transmission risk assessment and neonatal prophylaxis: a cluster randomized trial
Source: J Int AIDS Soc. 2025 Jul 28;28(8):e70021. doi: 10.1002/jia2.70021 (PMC12302277; doi:10.1002/jia2.70021)
Supplement: Supplementary file 1 — Table S1: Frequency and proportion of reasons for vertical HIV transmission high‐risk classification and Viral Load (VL) results performed at the point‐of‐care (intervention) versus retrospectively at the central laboratory (control). Infants who could be downgraded to low‐risk status based on VL results alone are indicated in yellow, infants categorized as high‐risk with maternal VL ≥1000 copies/ml at delivery are shown in green. Table S2: Frequency and proportion of HIV‐positive infants with high‐risk criteria for vertical HIV transmission between week 4 and 16 of life by study arm and country according to information available to health providers at delivery. [file JIA2-28-e70021-s001.docx]

**Supplemental Information**

**Table S1:** Frequency and proportion of reasons for vertical HIV transmission high-risk classification and Viral Load (VL) results performed at the point-of-care (intervention) versus retrospectively at the central laboratory (control). Infants who could be downgraded to low-risk status based on VL results alone are indicated in yellow, infants categorized as high-risk with maternal VL ≥1000 copies/ml at delivery are shown in green.


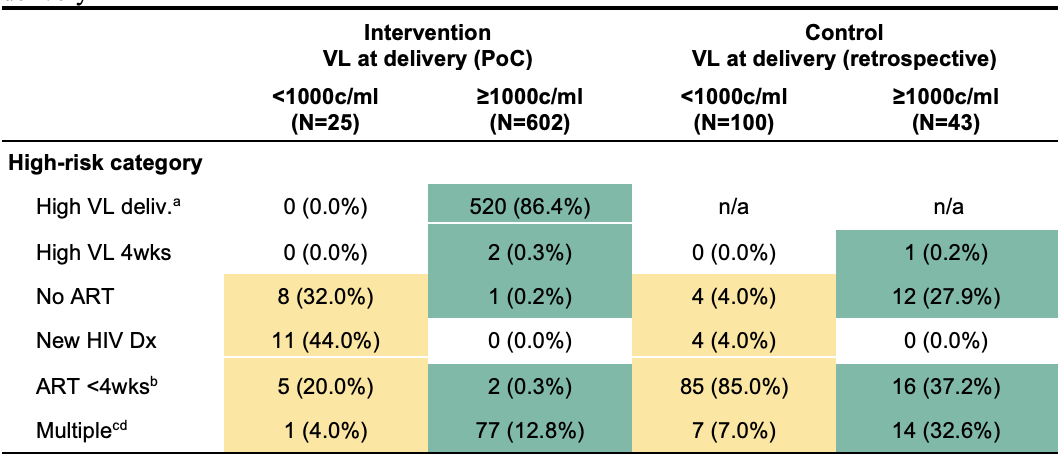


^a^In the intervention arm, Point of Care Viral Load (PoC VL) was performed at the site and immediately available during the delivery encounter; in the control arm, Viral Load (VL) was measured at the centralized laboratory with results available at a subsequent visit.

^b^N=3 no VL results available

^c^Two or more criteria for high-risk status. Only one mother in the intervention arm in Mozambique did not have VL ≥1000 copies/ml at delivery as one of the criteria for high-risk status.

^d^N=1 no VL results available

PoC: Point of Care

VL: Viral Load

**Table S2:** Frequency and proportion of HIV-positive infants with high-risk criteria for vertical HIV transmission between week 4 and 16 of life by study arm and country according to information available to health providers at delivery.


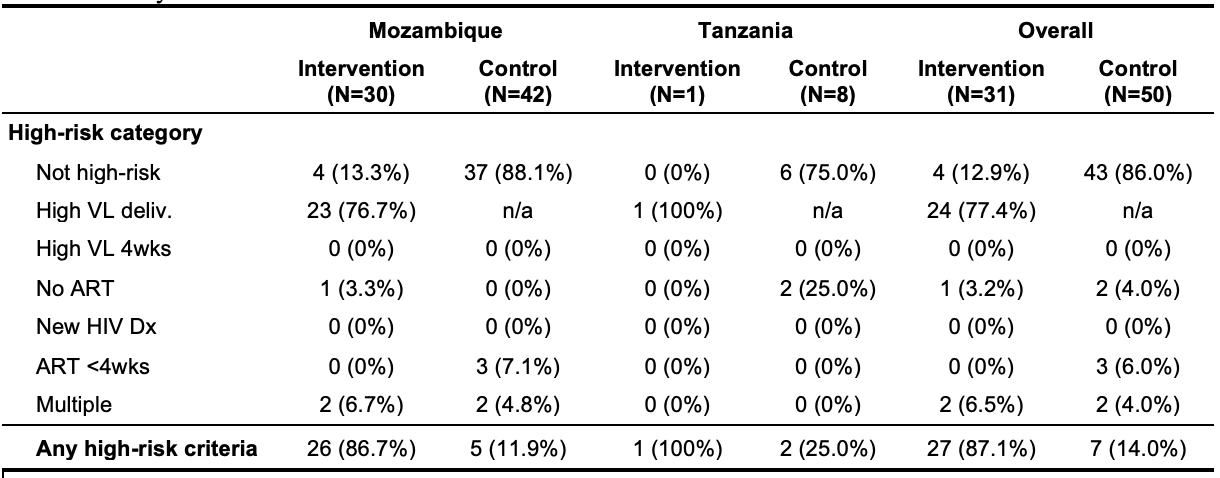


^a^In the intervention arm, Point of Care Viral Load (PoC VL) was performed at the site and immediately available during the delivery encounter; in the control arm, Viral Load (VL) was measured at the centralized laboratory with results available at a subsequent visit.

^b^Two or more criteria for high-risk status. Only one mother in the intervention arm in Mozambique did not have VL ≥1000 copies/ml at delivery as one of the criteria for high-risk status.
